# Cancer type-specific modulation of mitochondrial haplogroups in breast, colorectal and thyroid cancer

**DOI:** 10.1186/1471-2407-10-421

**Published:** 2010-08-12

**Authors:** Hezhi Fang, Lijun Shen, Tao Chen, Jing He, Zhinan Ding, Jia Wei, Jianchun Qu, Guorong Chen, Jianxin Lu, Yidong Bai

**Affiliations:** 1 Zhejiang Provincial Key Laboratory of Medical Genetics, Wenzhou Medical College, Wenzhou 325035, China; 2 Department of pathology of the First Affiliated Hospital, Wenzhou Medical College, Wenzhou 325000, China; 3 Department of Cellular and Structural Biology, University of Texas Health Science Center at San Antonio, San Antonio, TX 78229, USA

## Abstract

**Background:**

Mitochondrial DNA (mtDNA) haplogroups and single nucleotide polymorphisms (mtSNP) have been shown to play a role in various human conditions including aging and some neurodegenerative diseases, metabolic diseases and cancer.

**Methods:**

To investigate whether mtDNA haplogroups contribute to the occurrence of cancer in a specific Chinese population, we have carried out a comprehensive case-control study of mtDNA from large cohorts of patients with three common cancer types, namely, colorectal cancer (n = 108), thyroid cancer (n = 100) and breast cancer (n = 104), in Wenzhou, a southern Chinese city in the Zhejiang Province.

**Results:**

We found that patients with mtDNA haplogroup M exhibited an increased risk of breast cancer occurrence [OR = 1.77; 95% CI (1.03-3.07); P = 0.040], and that this risk was even more pronounced in a sub-haplogroup of M, D5 [OR = 3.11; 95%CI (1.07-9.06); p = 0.030]. In spite of this, in patients with breast cancer, haplogroup M was decreased in the metastatic group. On the other hand, our results also showed that haplogroup D4a was associated with an increased risk of thyroid cancer [OR = 3.00; 95%CI (1.09-8.29); p = 0.028]. However, no significant correlation has been detected between any mtDNA haplogroups and colorectal cancer occurrence.

**Conclusion:**

Our investigation indicates that mitochondrial haplogroups could have a tissue-specific, population-specific and stage-specific role in modulating cancer development.

## Background

Mitochondria, known as the cellular power plants, also regulate cell death and cell proliferation[1]. Mitochondria are under dual genome control. Human mtDNA encodes 13 essential subunits of the oxidative phosphorylation (OXPHOS) system as well as 2 rRNAs and 22 tRNAs used in mitochondrial translation. Alterations in mtDNA including both mutations and polymorphisms have the potential of changing the capacity of mitochondrial function. In particular, changes in oxidative phosphorylation resulting from mitochondrial dysfunction have long been hypothesized to be involved in tumorigenesis. To explain the fact that cancer cells were high in fermentation and low in respiration, Warburg proposed that cancer originated from a non-neoplastic cell which adopted anaerobic metabolism as a means of survival after injury to its respiratory system[2], which led to the notion that tumors were initiated by persistent damage to the mitochondria[3,4]. Supporting such idea, changes in the number, shape, and function of mitochondria have been reported in various cancers [5]. Interestingly, abnormal mtDNA was observed in leukemic myeloid cells using an electron microscope[6,7] long before DNA sequencing technology was available. Subsequently, mutations in both the non-coding and coding regions of the mtDNA have been identified in various types of cancer [8-10].

mtDNA is predominantly maternally inherited, and largely lacks recombination[11]. A human mtDNA haplogroup is defined by unique sets of mtDNA polymorphisms, reflecting mutations accumulated by a discrete maternal lineage[12]. The haplogroups are associated with region-specific mtDNA sequence variation as a result of genetic drift and/or adaptive selection for an environment-favored mitochondrial function[13,14]. Difference in redox signaling as a consequence of haplogroup-associated oxidative phosphorylation capacity has been suggested as the molecular mechanism involved in the haplogroups-associated phenotypes[15,16]. The haplogroup association studies have been used to investigate the effect of mtDNA variants on various complex conditions such as aging[17,18], metabolic diseases[19,20], neurodegenerative diseases [21,22], infectious diseases [23,24] and cancer[3,8,9]. In particular, haplogroups D4a, D5 and D4b2b were reported to be increased in centenarians in Japanese [25,26], while D4 was found to be enriched in female and N9 and M9 decreased in a Chinese population in Rugao area[17]. mtSNP at 10398, which is a major diagnostic site for macro-haplogroups M and N in Chinese population[27,28], has been implicated in longevity[29], Parkinson's disease[30], and breast cancer in various populations in some seemingly conflicting reports[31-34]. Interestingly, while reported as enriched in group exhibits longevity, D4a and D5a were observed to increase susceptibility in a Chinese population to esophageal carcinoma[35].

To assess the possible contribution of mtDNA haplogroup-specific polymorphisms to the prevalence of cancer in a southern Chinese population, we performed a case-control study of patients with three of the local most common types of cancer, breast cancer, thyroid cancer and colorectal cancer in Wenzhou, Zhejiang Province of China.

## Methods

### Subjects

The number and age information (in years) for the cancer patients used in this study: 108 colorectal cancer patients (mean ± SD 62.98 ± 12.63, median = 64, range 30-88), 104 breast cancer patients (mean ± SD 51.6 ± 10.29, median = 51, range 27-79) and 100 thyroid cancer patients (mean ± SD 45.98 ± 12.59, median = 46, range 20-84). The patients were recruited when they were admitted for surgery at the First Affiliated Hospital of Wenzhou Medical College from May, 2007 to November, 2008. Histological confirmations of each cancer type were carried out immediately following each surgery. Three sets of age, gender and geographically matched control subjects who had a cancer-free history, and had no other known diseases which could be associated with mitochondrial defects were also recruited at the Physical Examination Center of the same hospital from October, 2008 to December, 2008. The numbers and ages (in years) of controls for each study are as follows: n = 124 (mean ± SD 60.15 ± 9.3, median = 60, range 37-84) for the colorectal cancer study, n = 114 (mean ± SD 53.44 ± 11.66, median = 52, range 27-84) for the breast cancer study, and n = 138 (mean ± SD 47.96 ± 6.64, median = 49, range 27-62) for the thyroid cancer study. Informed consents were obtained from all patients and controls according to the regulations set forth by the ethical committee of Wenzhou Medical College.

### mtDNA sequencing

About 2 ml venous blood was collected from each sample before surgery and any drug treatment. Total DNA was extracted using a standard phenol-chloroform method as described previously[36]. The sequences of two pairs of primers designed to amplify the mtDNA D-loop and ND3+tRNA^arg^+ND4L region were as follows, L15792F: TCATTGGACAAGTAGCATCC, H794R: AGGCTAAGCGTTTTGAGCTG and L9967F: TCTCCATCTATTGATGAGGGTCT, H10858R: AATTAGGCTGTGGGTGGTTG[37]. PCR was performed on a Thermal Cycler 170-9703 PCR machine (BIO-RAD, USA). The PCR conditions were as follows: pre-denaturation at 95°C for 5 min, then 35 cycles of [94°C for 30 s, 57°C for 35 s, 72°C for 1 min], and a final extention at 72°C for 4 min. The PCR products were purified using the Agarose Gel DNA Fragment Recovery Kit Ver.2.0 (TaKaRa, Japan) and subsequently sequenced on an ABI Prism 3730 sequence analyzer.

### mtDNA haplogroup and mtSNPs analysis

To assign a mtDNA haplogroup to each sample, all sequences were compared with the revised Cambridge Reference Sequence (rCRS)[38] and aligned using the CodonCode Aligner 3.0.1 (CodonCode Corporation, USA)[37] software program. Based on the most recent refined East Asian mitochondrial haplogroup tree [27,28,39], the initial assignment was performed with the sequencing information from the D-loop, ND3 and ND4L regions. When necessary, additional information was obtained by restriction fragment length polymorphism (RFLP) analysis at sites: 663 (*Hae*III), 3394 (*Hae*III), 4833 (*Hha*I), 5178 (*Alu*I) and 9824 (*Hin*fI). With some samples, the 9 bp deletion at the COII-tRNA^lys ^junction was also detected to further identification.

### Statistical analysis

All statistical analyses were performed using SPSS software (version 13.0) (SPSS Inc., Chicago, IL, USA). The Pearson chi-square test was used to analyze the relationship between the prevalence of haplogroups and different cancer features. Binary logistic regression analysis was also carried out to determine the contributions of haplogroup and other risk factors to cancer features.

## Results

### Macro-haplogroups M and N on breast cancer

**Figure 1:**
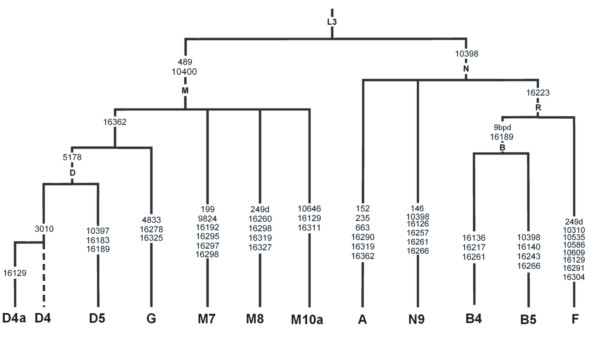
**Classification of 12 mtDNA haplogroups in subjects of three case-control studies**. The defining sites[38] (as compared with the revised CRS) utilized in this study are listed in the branches. "d" indicates a deletion; "9 bpd" indicates a 9 bp deletion in the intergenic mtDNA region between nucleotides 8195-8316[53].

Macro-haplogroups M and N are major descendents of L3, a superhaplogroup and an "African Eve" which shapes all various sub-lineages out of Africa[12,40]. These specific haplogroups could be identified by the polymorphisms at positions 10400 and 10398 in the Chinese Han population, where M is defined by T at 10400 and likely exhibits G at 10398 whereas N has A at 10398 as in the Cambridge Standard sequence[28] (Figure.1). It was reported that haplogroup N was associated with an increased risk of breast cancer in the Indian population[33]. Interestingly, 10398A in African-American[31] and 10398G in European-American[34] populations were both reported to correlate with increased breast cancer risks.

In a case-control study (CCS) conducted with breast cancer patients in a southern Chinese population, we tested the correlation between macro-haplogroups M and N and breast cancer incidence. We found that haplogroup M posed a significant risk for breast cancer (OR = 1.77; 95%CI 1.03-3.07; P = 0.040), whereas haplogroup N was associated with a decreased incidence (OR = 0.56; 95%CI 0.33-0.98; P = 0.040) (Table 1). Following age-adjustment of the data in a binary logistic regression analysis, the results became even more significant. Haplogroup M had a higher occurrence in breast cancer patients (OR = 1.84; 95%CI 1.07-3.20; P = 0.029), whereas haplogroup N was decreased in frequency (OR = 0.54; 95%CI 0.31-0.94; P = 0.029). We then examined the mtSNP at position 10398, and found that 10398G was correlated with an increased incidence of breast cancer (OR = 1.77; 95%CI 1.00-3.14; P = 0.050), and 10398A showed a protection effect (OR = 0.56; 95%CI 0.32-1.00; P = 0.050) (Table 1). A stronger influence was revealed after a binary logistic regression analysis for the age-adjustment for both 10398G (OR = 1.98; 95%CI 1.11-3.53, P = 0.021) and 10398A (OR = 0.51, 95%CI 0.28-0.90, P = 0.021).

**Table 1:** Effect of macro haplogroup M and N on breast cancer occurrence

Sample	Value	Haplogroups		*P *value	OR (95%CI)	10398		*P *value	OR (95%CI)
		M(n)	N(n)			G	A		
Cases	104	69	35	0.040*	1.77(1.03-3.07)	76	28	0.050*	1.77(1.00-3.14)
Controls	114	60	54			69	45		

*P *values were estimated by "chi-square test"; (*) indicated statistical significant (P < 0.05). OR indicates odds ratio; 95% CI, 95%confidence interval.

We also analyzed some clinical characteristics and other risk factors for breast cancer in the breast cancer patients divided among macro-haplogroups M and N. As shown in Table 2, age is not a contributing factor (P = 0.838) to the differences we observed in breast cancer patients with macro-haplogroups M and N. Similarly, we did not detect a significant difference in Body Mass Index (BMI) between these patients. Further investigation of the expression of progesterone receptor (PR) and estrogen receptor (ER) failed to reveal any difference between the M and N haplogroups. However, surprisingly we found an enriched presentation of the N haplogroup in patients with metastatic breast cancer (P = 0.044). After adjusting with co-variants like age, BMI, PR and ER expression, N still exhibited a significantly higher occurrence in the metastatic group (OR = 0.39; 95%CI 0.17-0.94; P = 0.036).

**Table 2:** Stratification of clinical characteristics in breast cancer cases with haplogroup M and N

Variable	Value	Haplogroups		*P *value
			
		M(n)	N(n)	
Cancer metastasis				0.044*
yes	34	18	16	
No	70	51	19	
Progesterone receptor (PR)				0.818
PR positive	64	43	21	
PR negative	40	26	14	
Estrogen receptor (ER)				0.932
ER positive	63	42	21	
ER negative	41	27	14	
Age				0.838
≤50	49	33	16	
>50	55	36	19	
BMI				0.832
18-25	55	37	18	
>25 or <18	49	32	17	

*P *values were estimated by "chi-square test"; (*) indicated statistical significant (P < 0.05). BMI, body mass index; BMI ranging18-25 is considered as normal.

### Haplogroup analysis in breast cancer, colorectal cancer and thyroid cancer patients

We furthered our investigation by performing a more detailed sub-haplogroup analysis. As described in the Materials and Methods section, based on the mtSNPs shown in Figure 1, we were able to assign 104 breast cancer patients and 114 controls to 12 sub-haplogroups. As shown in Table 3, we found haplogroup D5, a sub-haplogroup of D under M (Figure. 1), displayed a significantly higher frequency in breast cancer patients compared with control subjects (OR = 3.11; 95%CI 1.07-9.06; P = 0.030). This significant correlation remained after age-adjustment of the data in binary logistic regression analysis (OR = 3.13; 95%CI: 1.07-9.13; P = 0.037).

**Table 3:** Effect of haplogroups on breast, colorectal and thyroid cancer occurrences

Haplogroup	CCS of breast cancer			*P *value	CCS of colorectal cancer			*P *value	CCS of thyroid cancer			*P *value
	Controls	Cases	OR		Controls	Cases	OR		Controls	Cases	OR	
	(N = 114)	(N = 104)	(95%CI)		(N = 125)	(N = 108)	(95%CI)		(N = 138)	(N = 100)	(95%CI)	
**M**	60	69	1.77(1.03-3.07)	0.040*	61	53	1.01(0.60-1.69)	0.967	70	55	1.19(0.71-1.99)	0.514
D	26	28	1.25(0.67-2.31)	0.482	32	26	0.92(0.51-1.67)	0.788	34	33	1.51(0.85-2.66)	0.157
D4	21	15	0.75(0.36-1.54)	0.427	23	19	0.95(0.48-1.85)	0.873	26	27	1.59(0.86-2.94)	0.135
D4a	6	4	0.72(0.20-2.63)	0.617	9	6	0.76(0.26-2.20)	0.610	6	12	3.00(1.09-8.29)	0.028*
D5	5	13	3.11(1.07-9.06)	0.030*	9	7	0.89(0.32-2.49)	0.829	8	6	1.04(0.35-3.09)	0.948
G	2	7	4.04(0.82-19.91)	0.065	3	0	-	0.105	3	1	0.46(0.05-4.44)	0.487
M8	8	15	2.23(0.91-5.51)	0.075	8	9	1.33(0.49-3.58)	0.571	11	6	0.74(0.26-2.06)	0.560
M7	16	8	0.51(0.21-1.25)	0.135	13	9	0.78(0.32-1.91)	0.591	15	11	1.01(0.44-2.31)	0.975
M10a	2	5	2.83(0.54-14.90)	0.202	1	3	3.54(0.36-34.57)	0.246	3	0	-	0.138
**N**												
A	10	4	0.42(0.13-1.37)	0.138	10	9	1.05(0.41-2.68)	0.926	11	9	1.14(0.46-2.87)	0.778
N9	3	4	1.48(0.32-6.78)	0.611	5	7	1.66(0.51-5.40)	0.393	4	5	1.76(0.46-6.74)	0.402
R	41	27	0.62(0.35-1.12)	0.111	48	36	0.80(0.47-1.37)	0.422	52	27	0.61(0.35-1.07)	0.084
B	23	14	0.69(0.33-1.44)	0.322	24	20	0.96(0.50-1.85)	0.895	26	16	0.82(0.41-1.63)	0.570
B4	16	8	0.51(0.21-1.25)	0.135	17	9	0.58(0.25-1.36)	0.203	19	9	0.62(0.27-1.43)	0.260
B5	7	5	0.77(0.24-2.51)	0.667	7	11	1.91(0.71-5.12)	0.191	7	7	1.41(0.48-4.15)	0.533
F	13	11	0.92(0.39-2.15)	0.846	19	16	0.97(0.47-2.00)	0.935	22	11	0.65(0.30-1.41)	0.276

*P *values were estimated by "chi-square test"; (*) indicated statistical significant (P < 0.05). OR indicates odds ratio; 95% CI, 95%confidence interval.

To determine if the coincidence of a specific mtDNA haplogroup with breast cancer indicates a general role in tumorigenesis in the southern Chinese population, we extended our studies to two other common cancer types in the Wenzhou area, colorectal cancer and thyroid cancer. Similar case-control studies were conducted on colorectal cancer and thyroid cancer patients. As shown in Table 3, unlike what we observed for breast cancer, there was no correlation between haplogroups M, N or D5 and occurrences of these cancers. However, our results revealed that haplogroup D4a, a sub-haplogroup of D4, which shares the same ancestry of D and M with D5 (Figure. 1), had a significantly higher frequency in thyroid cancer patients relative to control subjects (OR = 3.00; 95%CI 1.087-8.29; P = 0.028). After adjusting the data for age and gender in binary logistic regression analysis, the observed difference between cases and controls remained significant (OR = 3.18; 95%CI 1.11-9.11; P = 0.031). Nevertheless, no significant correlation has been detected between any mtDNA haplogroups and colorectal cancer occurrence (Table 3).

### mtSNP and combinations of mtSNPs in breast cancer, colorectal cancer and thyroid cancer patients

To determine the incidence of individual mtSNPs within haplogroups D4a and D5 in breast or thyroid cancer patient samples, we performed a stepwise investigation of the formation of the D5 and D4a sub-haplogroups and examined their potential correlation with cancer occurrences. As shown in Table 4, the combination of 10400T and 489C, which mostly determined macro-haplogroup M in the Chinese Han population, was associated with an increased breast cancer incidence. An additional 16362C, which mostly further defined the D plus G haplogroups, somehow negated such a correlation, whereas 10397G, which helped to determine D5, restored the observed association with an increased risk of breast cancer. Individually, only 10398G (people with 10397G all belong to this group) exhibited an increased frequency in breast cancer patients relative to control subjects. For thyroid cancer, the combination of 16362C and 16129A, both in the highly variable area of the D-loop region, exhibited a significant correlation with cancer incidence.

**Table 4:** Effect of D4a and D5 determining mtSNPs on breast cancer and thyroid cancer

mtSNP	CCS of breast cancer			*P *value	CCS of thyroid cancer			*P *value
				
	Controls	Cases	OR		Controls	Cases	OR	
	(N = 114)	(N = 104)	(95%CI)		(N = 138)	(N = 100)	(95%CI)	
C10400T (T489C)	60	69	1.77(1.03-3.07)	0.040*	70	55	1.19(0.71-1.99)	0.514
T16362C+C10400T(T489C)	30	35	1.42(0.79-2.54)	0.237	38	36	1.48(0.85-2.57)	0.164
A10397G+T16362C+C10400T(T489C)	4	13	3.93(1.24-12.46)	0.013*	6	6	1.40(0.44-4.49)	0.565
G16129A+T16362C+C10400T(T489C)	6	4	0.72(0.20-2.63)	0.617	6	12	3.00(1.09-8.29)	0.028*
A10397G+C10400T(T489C)	5	13	3.11(1.07-9.06)	0.030*	8	6	1.04(0.35-3.09)	0.948
A10397G	5	13	3.11(1.07-9.06)	0.030*	8	6	1.04(0.35-3.09)	0.948
A10398G	69	76	1.77(1.00-3.14)	0.050*	74	66	1.08(0.64-1.83)	0.766
T16362C	37	37	1.15(0.66-2.01)	0.627	50	43	1.33(0.78-2.25)	0.291
G16129A	27	31	1.53(0.84-2.78)	0.165	28	28	1.53(0.84-2.79)	0.166

*P *values were estimated by "chi-square test"; (*) indicated statistical significant (P < 0.05). OR indicates odds ratio; 95% CI, 95% confidence interval.

In addition, all of the mtSNPs in the D-loop region, which were not utilized to determine the haplogroups and identified with a frequency of more than 5% in this study, were also analyzed. As shown in Table 5, none of them displayed significant association with the occurrence of either of the cancer types.

**Table 5:** Effect of non-haplogroup determining mtSNPs on breast cancer, colorectal cancer and thyroid cancer

	CCS of breast cancer				CCS of colorectal cancer				CCS of thyroid cancer			
						
mtSNPs	Controls	Cases	OR	*P *value	Controls	Cases	OR	P value	Controls	Cases	OR	*P *value
	(N = 114)	(N = 104)	(95%CI)		(N = 125)	(N = 108)	(95%CI)		(N = 138)	(N = 100)	(95%CI)	
T16140C	11	7	0.68(0.25-1.81)	0.434	13	14	1.28(0.58-2.87)	0.542	13	7	0.72(0.28-1.89)	0.507
A16182C	11	18	1.96(0.88-4.37)	0.096	18	11	0.67(0.30-1.50)	0.331	15	13	1.23(0.56-2.71)	0.615
A16183C	29	28	1.08(0.59-1.98)	0.803	36	22	0.63(0.34-1.16)	0.138	32	25	1.10(0.61-2.01)	0.747
T16189C	38	35	1.01(0.58-1.78)	0.960	42	36	0.99(0.57-1.71)	0.966	46	26	0.70(0.40-1.24)	0.224
T16217C	16	11	0.72(0.32-1.64)	0.439	17	9	0.58(0.25-1.36)	0.203	19	9	0.62(0.27-1.43)	0.260
C16223T	69	68	1.23(0.71-2.14)	0.458	73	66	1.12(0.66-1.89)	0.674	83	62	1.08(0.64-1.83)	0.772
T16298C	10	12	1.36(0.56-3.29)	0.498	10	14	1.71(0.73-4.03)	0.214	12	6	0.67(0.24-1.85)	0.438
T16304C	11	13	1.33(0.57-3.13)	0.502	23	14	0.66(0.32-1.36)	0.257	28	13	0.59(0.29-1.20)	0.142
T16311C	20	17	0.92(0.45-1.87)	0.814	23	13	0.61(0.29-1.27)	0.180	19	9	0.62(0.27-1.43)	0.260
G16319A	15	13	0.94(0.43-2.09)	0.885	11	18	2.07(0.93-4.61)	0.070	17	16	1.36(0.65-2.83)	0.417
T146C	16	17	1.20(0.57-2.51)	0.634	13	17	1.61(0.74-3.49)	0.225	20	10	0.66(0.29-1.47)	0.303
C150T	28	33	1.43(0.79-2.59)	0.239	33	25	0.84(0.46-1.53)	0.567	33	22	0.90(0.49-1.66)	0.730
T152C	20	23	1.34(0.68-2.61)	0.397	27	26	1.15(0.62-2.13)	0.653	33	31	1.43(0.80-2.55)	0.224
249delA	20	19	1.05(0.53-2.10)	0.889	25	23	1.08(0.57-2.04)	0.807	31	13	0.52(0.25-1.05)	0.063
524delAC	42	27	0.60(0.34-1.07)	0.084	43	33	0.84(0.48-1.46)	0.532	52	42	1.20(0.71-2.0)	0.501

*P *values were estimated by "chi-square test"; (*) indicated statistical significant (P < 0.05). OR indicates odds ratio; 95% CI, 95% confidence interval.3

## Discussion

In this study, a total of 312 cancer patients (104 with breast cancer, 108 with colorectal cancer and 100 with thyroid cancer) and their matched control subjects were analyzed for the mitochondrial haplogroups and some related mtSNPs. In the Wenzhou area, breast cancer ranks number one in cancer occurrence among females, as in the other regions of the world. Thyroid cancer ranks number one in the age group of 15-34, and colon cancer tops liver, lung and stomach cancers, in cancer patients under 15 years old, both indicating a genetic predisposition[41]. An age and regional matched study increased our chances of discovering a contribution from mtDNA.

The first finding of our study is that macro-haplogroup M has an increased frequency in breast cancer patients relative to controls, and intriguingly, breast cancer patients in macro-haplogroup N are more likely to exhibit metastatic tumors. Previously, a similar CCS with 124 sporadic breast cancer patients and 273 controls, together with analysis of 2334 individuals belonging to 18 regions in India, led to the proposition that mtSNP 10398A imparted haplogroup N with an increased risk for breast cancer[33].

A10398G is probably one of the best studied mtSNPs, in particular with respect to its potential effect on tumorigenesis. Noticeably, A10398G changes a threonine residue (A allele) to an alanine (G allele) at the C-terminus of the ND3 subunit of respiratory complex I. Using the cybrids system, it was reported that the closely linked 8701A/10398A mtSNPs were associated with a lowered mitochondrial matrix pH, and at the same time an increased basal level of mitochondrial calcium and cytosolic calcium response to histamine[42]. A series of epidemiological investigations of neurodegenerative diseases like Parkinson's disease[30], Alzheimer's disease[43], and amyotropic lateral sclerosis (ALS)[44] suggested that the 10398A allele is associated with the degenerative phenotype.

In a large population-based CCS with 654 cases and 605 controls, African-American women with the 10398A allele were shown to have a significant risk of invasive breast cancer, but such a correlation was not observed in white women[31]. In a separate CCS with 156 unrelated European-American women with familial breast cancer and 260 controls, 10398G was associated with an increased risk of breast cancer[34]. An independent investigation carried out with a Polish breast cancer population also suggested 10398G as an inherited predisposition factor for the development of breast cancer[45]. However, other studies of either African-American females[46] or Spain and Canary Islands women[47] failed to confirm a role for mtSNP at 10398 in breast cancer development. Nevertheless, 10398G was found as a risk factor in oral cancer among Indian smokers[48].

To reconcile these seemingly conflicting results, based on our own data, we proposed that the combination of certain mtDNA haplogroups or mtSNPs (in this case, 10398A or G) with other nuclear encoded factors (some potentially tissue-specific), could play a role in tumorigenesis. The net result could be either alteration of calcium or redox signaling. It is also important to note that the enhanced generation of reactive oxygen species (ROS), which has been suggested by several investigators as a likely underlying mechanism by which mitochondrial haplogroups and mtSNPs play a role in cancer development[8,25,31,34], could both activate an oncogenic pathway which would lead to being a risk factor for cancer occurrence, or activate the apoptotic reaction, which could display a protective effect in the late stages of cancer development. Other determining factors in this process include the cellular thresholds required to activate those pathways, which also could be tissue-specific.

Another major finding of our studies is that mtDNA haplogroups D4a and D5 were associated with an increased risk of breast cancer and thyroid cancer, respectively, in the southern Chinese population. In a previous investigation on longevity in a Japanese population, D4a and D5 were found to be enriched in centenarians[25]. It was further hypothesized that the replacement of Ile78Thr in the cytochrome b subunit of respiratory complex III in D4a, and Ile278Val in ND2 and Ile423Val in ND4 of complex I in D5 could play roles in modulating ROS production[25,26]. It is worth noting that aging and cancer could both result from the deregulation of cell homeostasis[49]. The regulation of homeostasis is mostly achieved by the balanced control of cell death and cell proliferation. Thus it is possible that the long lifespan observed to be associated with D4a and D5 could be achieved at the expense of higher cancer incidence. Consistent with this notion, it was reported that D5a and D4a are risk factors of esophageal cancer in the Chaoshan and Taihang Mountain areas of southern China[35]. In fact, most patients and control subjects identified as D5 in our study are indeed D5a, as most of the D5 sub-haplogroup individuals in southern China could be further designated as D5a. In another study, haplogroup D was suggested as a likely risk factor for endometrial cancer in southwestern China[50].

In addition to 10398 variants, the functional implications of the haplopgroup J defining mtSNP C295T in the D-loop region were also examined. It was reported that C295T was associated with an increased binding of TFAM (transcription factor A, mitochondrial), and as a result, cybrids with haplogroup J had a significantly increased mtDNA copy number compared with those carrying haplogroup H[51]. It is possible that the combination of 16362C and 16129A in the D-loop region observed in our study could have an effect on mtDNA replication and/or transcription as well.

Furthermore, a major finding of our study suggested that mitochondrial macro-haplogroup M or mtSNP 10398G could exert different effects under different nuclear background. This is consistent with results of our previous study in which we found that mtDNA mutations, probably mediated by ROS and apoptosis, can play different roles in different stages of cancer development[52]. The potential regulation of tumorigenesis by mtDNA haplogroups or mtSNPs is likely facilitated by nuclear encoded factors, as evidenced by the tissue-specific and population-specific features of mtDNA haplogroups. To test this hypothesis, it would be necessary to extend our investigation to include a greater diversity of populations and cancer types, and more importantly complementary functional assays.

Finally, based on results obtained from this study, an extended investigation including larger cohort and with more cancer types would be warranted to reveal further the interaction between mitochondrial and nuclear genome in cancer cells.

## Conclusions

We found macro-haplogroup M and its sub-haplogroup D5 exhibited an increased risk of breast cancer occurrence but haplogroup M was decreased in the metastatic group. On the other hand, haplogroup D4a was associated with an increased risk of thyroid cancer, while no significant correlation has been detected between any mtDNA haplogroups and colorectal cancer occurrence. Our data indicate that mitochondrial haplogroups could have a tissue-specific, population-specific and stage-specific role in modulating cancer development.

## Competing interests

The authors declare that they have no competing interests.

## Authors' contributions

HF carried out the sequencing analysis. HF, LS, ZD, JW, JQ and GC collected samples and carried out the pathological analysis. HF, LS, TC and JH carried out the statistical analysis. YB, JL and HF conceived the study, participated in the design the experiments and drafted the manuscript. All authors read and approved the final manuscript

## Pre-publication history

The pre-publication history for this paper can be accessed here:

http://www.biomedcentral.com/1471-2407/10/421/prepub

## References

[B1] Wallace DC Mitochondria as chi Genetics 2008 179 2 727 735 10.1534/genetics.104.91769 18558648 PMC2429869

[B2] Warburg O On the origin of cancer cell Science 1956 123 309 314 10.1126/science.123.3191.309 13298683

[B3] Lu J Sharma LK Bai Y Implications of mitochondrial DNA mutations and mitochondrial dysfunction in tumorigenesis Cell Res 2009 19 7 802 815 10.1038/cr.2009.69 19532122 PMC4710094

[B4] Shen L Fang H Chen T He J Zhang M Wei X Xin Y Jiang Y Ding Z Ji J Evaluating mitochondrial DNA in cancer occurrence and development Ann N Y Acad Sci 1201 26 33 10.1111/j.1749-6632.2010.05635.x 20649535

[B5] Pedersen PL Tumor mitochondria and the bioenergetics of cnacer cells Proc Exp Tumor Res 1978 22 190 274 10.1159/000401202 149996

[B6] Clayton DA Vinograd J Circular dimer and catenate forms of mitochondrial DNA in human leukaemic leucocytes J Pers 1967 35 4 652 657 10.1038/216652a0 6082459

[B7] Clayton DA Vinograd J Complex mitochondrial DNA in leukemic and normal human myeloid cells Proc Natl Acad Sci USA 1969 62 4 1077 1084 10.1073/pnas.62.4.1077 5256408 PMC223617

[B8] Brandon M Baldi P Wallace DC Mitochondrial mutations in cancer Oncogene 2006 25 34 4647 4662 10.1038/sj.onc.1209607 16892079

[B9] Chatterjee A Mambo E Sidransky D Mitochondrial DNA mutations in human cancer Oncogene 2006 25 34 4663 4674 10.1038/sj.onc.1209604 16892080

[B10] Shen L Wei J Chen T He J Qu J He X Jiang L Qu Y Fang H Chen G Evaluating mitochondrial DNA in patients with breast cancer and benign breast disease J Cancer Res Clin Oncol10.1007/s00432-010-0912-x PMC11827960 20552226

[B11] Pakendorf B Stoneking M Mitochondrial DNA and human evolution Annu Rev Genomics Hum Genet 2005 6 165 183 10.1146/annurev.genom.6.080604.162249 16124858

[B12] Watson E Forster P Richards M Bandelt HJ Mitochondrial footprints of human expansions in Africa Am J Hum Genet 1997 61 3 691 704 10.1086/515503 9326335 PMC1715955

[B13] Johnson MJ Wallace DC Ferris SD Rattazzi MC Cavalli-Sforza LL Radiation of human mitochondria DNA types analyzed by restriction endonuclease cleavage patterns J Mol Evol 1983 19 3-4 255 271 10.1007/BF02099973 6310133

[B14] Ruiz-Pesini E Mishmar D Brandon M Procaccio V Wallace DC Effects of purifying and adaptive selection on regional variation in human mtDNA Science 2004 303 5655 223 226 10.1126/science.1088434 14716012

[B15] Tanaka M Takeyasu T Fuku N Li-Jun G Kurata M Mitochondrial genome single nucleotide polymorphisms and their phenotypes in the Japanese Ann N Y Acad Sci 2004 1011 7 20 10.1196/annals.1293.002 15126279

[B16] Wallace DC A mitochondrial paradigm of metabolic and degenerative diseases, aging, and cancer: a dawn for evolutionary medicine Annu Rev Genet 2005 39 359 407 10.1146/annurev.genet.39.110304.095751 16285865 PMC2821041

[B17] Cai XY Wang XF Li SL Qian J Qian DG Chen F Yang YJ Yuan ZY Xu J Bai Y Association of mitochondrial DNA haplogroups with exceptional longevity in a chinese population PLoS One 2009 4 7 e6423 10.1371/journal.pone.0006423 19641616 PMC2713402

[B18] Yao YG Kong QP Zhang YP Mitochondrial DNA 5178A polymorphism and longevity Hum Genet 2002 111 4-5 462 463 10.1007/s00439-002-0826-z 12384792

[B19] Fuku N Park KS Yamada Y Nishigaki Y Cho YM Matsuo H Segawa T Watanabe S Kato K Yokoi K Mitochondrial haplogroup N9a confers resistance against type 2 diabetes in Asians Am J Hum Genet 2007 80 3 407 415 10.1086/512202 17273962 PMC1821119

[B20] Tanaka M Fuku N Nishigaki Y Matsuo H Segawa T Watanabe S Kato K Yokoi K Ito M Nozawa Y Women with mitochondrial haplogroup N9a are protected against metabolic syndrome Diabetes 2007 56 2 518 521 10.2337/db06-1105 17259400

[B21] Ji Y Zhang AM Jia X Zhang YP Xiao X Li S Guo X Bandelt HJ Zhang Q Yao YG Mitochondrial DNA haplogroups M7b1'2 and M8a affect clinical expression of leber hereditary optic neuropathy in Chinese families with the m.11778G-->a mutation Am J Hum Genet 2008 83 6 760 768 10.1016/j.ajhg.2008.11.002 19026397 PMC2668067

[B22] Tanaka N Goto YI Akanuma J Kato M Kinoshita T Yamashita F Tanaka M Asada T Mitochondrial DNA variants in a Japanese population of patients with Alzheimer's disease Mitochondrion 2010 10 1 32 37 10.1016/j.mito.2009.08.008 19703591

[B23] Hendrickson SL Hutcheson HB Ruiz-Pesini E Poole JC Lautenberger J Sezgin E Kingsley L Goedert JJ Vlahov D Donfield S Mitochondrial DNA haplogroups influence AIDS progression AIDS 2008 22 18 2429 2439 10.1097/QAD.0b013e32831940bb 19005266 PMC2699618

[B24] Hendrickson SL Kingsley LA Ruiz-Pesini E Poole JC Jacobson LP Palella FJ Bream JH Wallace DC O'Brien SJ Mitochondrial DNA haplogroups influence lipoatrophy after highly active antiretroviral therapy J Acquir Immune Defic Syndr 2009 51 2 111 116 10.1097/QAI.0b013e3181a324d6 19339895 PMC2742970

[B25] Alexe G Fuku N Bilal E Ueno H Nishigaki Y Fujita Y Ito M Arai Y Hirose N Bhanot G Enrichment of longevity phenotype in mtDNA haplogroups D4b2b, D4a, and D5 in the Japanese population Hum Genet 2007 121 3-4 347 356 10.1007/s00439-007-0330-6 17308896

[B26] Bilal E Rabadan R Alexe G Fuku N Ueno H Nishigaki Y Fujita Y Ito M Arai Y Hirose N Mitochondrial DNA haplogroup D4a is a marker for extreme longevity in Japan PLoS One 2008 3 6 e2421 10.1371/journal.pone.0002421 18545700 PMC2408726

[B27] Kong QP Bandelt HJ Sun C Yao YG Salas A Achilli A Wang CY Zhong L Zhu CL Wu SF Updating the East Asian mtDNA phylogeny: a prerequisite for the identification of pathogenic mutations Hum Mol Genet 2006 15 13 2076 2086 10.1093/hmg/ddl130 16714301

[B28] Yao YG Kong QP Bandelt HJ Kivisild T Zhang YP Phylogeographic differentiation of mitochondrial DNA in Han Chinese Am J Hum Genet 2002 70 3 635 651 10.1086/338999 11836649 PMC384943

[B29] Niemi AK Moilanen JS Tanaka M Hervonen A Hurme M Lehtimaki T Arai Y Hirose N Majamaa K A combination of three common inherited mitochondrial DNA polymorphisms promotes longevity in Finnish and Japanese subjects Eur J Hum Genet 2005 13 2 166 170 10.1038/sj.ejhg.5201308 15483642

[B30] van der Walt JM Nicodemus KK Martin ER Scott WK Nance MA Watts RL Hubble JP Haines JL Koller WC Lyons K Mitochondrial polymorphisms significantly reduce the risk of Parkinson disease Am J Hum Genet 2003 72 4 804 811 10.1086/373937 12618962 PMC1180345

[B31] Canter JA Kallianpur AR Parl FF Millikan RC Mitochondrial DNA G10398A polymorphism and invasive breast cancer in African-American women Cancer Res 2005 65 17 8028 8033 16140977 10.1158/0008-5472.CAN-05-1428

[B32] Mims MP Hayes TG Zheng S Leal SM Frolov A Ittmann MM Wheeler TM Prchal JT Mitochondrial DNA G10398A polymorphism and invasive breast cancer in African-American women Cancer Res 2006 66 3 1880 author reply 1880-1881. 10.1158/0008-5472.CAN-05-3774 16452251 PMC6181229

[B33] Darvishi K Sharma S Bhat AK Rai E Bamezai RN Mitochondrial DNA G10398A polymorphism imparts maternal Haplogroup N a risk for breast and esophageal cancer Cancer Lett 2007 249 2 249 255 10.1016/j.canlet.2006.09.005 17081685

[B34] Bai RK Leal SM Covarrubias D Liu A Wong LJ Mitochondrial genetic background modifies breast cancer risk Cancer Res 2007 67 10 4687 4694 10.1158/0008-5472.CAN-06-3554 17510395

[B35] Li XY Su M Huang HH Li H Tian DP Gao YX mtDNA evidence: genetic background associated with related populations at high risk for esophageal cancer between Chaoshan and Taihang Mountain areas in China Genomics 2007 90 4 474 481 10.1016/j.ygeno.2007.06.006 17689918

[B36] Deng JH Li Y Park JS Wu J Hu P Lechleiter J Bai Y Nuclear suppression of mitochondrial defects in cells without the ND6 subunit Mol Cell Biol 2006 26 3 1077 1086 10.1128/MCB.26.3.1077-1086.2006 16428459 PMC1347011

[B37] Rieder MJ Taylor SL Tobe VO Nickerson DA Automating the identification of DNA variations using quality-based fluorescence re-sequencing: analysis of the human mitochondrial genome Nucleic Acids Res 1998 26 4 967 973 10.1093/nar/26.4.967 9461455 PMC147367

[B38] Andrews RM Kubacka I Chinnery PF Lightowlers RN Turnbull DM Howell N Reanalysis and revision of the Cambridge reference sequence for human mitochondrial DNA Nat Genet 1999 23 2 147 10.1038/13779 10508508

[B39] Kivisild T Tolk HV Parik J Wang Y Papiha SS Bandelt HJ Villems R The emerging limbs and twigs of the East Asian mtDNA tree Mol Biol Evol 2002 19 10 1737 1751 12270900 10.1093/oxfordjournals.molbev.a003996

[B40] Torroni A Achilli A Macaulay V Richards M Bandelt HJ Harvesting the fruit of the human mtDNA tree Trends Genet 2006 22 6 339 345 10.1016/j.tig.2006.04.001 16678300

[B41] Wenqiu Zheng ZZ An Analysis Of Cancer Incidencee In 2005 In Lucheng Discricr, Wenzhou City Journal Of Chinese Cancer 2007 16 5 306 308

[B42] Kazuno AA Munakata K Nagai T Shimozono S Tanaka M Yoneda M Kato N Miyawaki A Kato T Identification of mitochondrial DNA polymorphisms that alter mitochondrial matrix pH and intracellular calcium dynamics PLoS Genet 2006 2 8 e128 10.1371/journal.pgen.0020128 16895436 PMC1534079

[B43] Shoffner JM Brown MD Torroni A Lott MT Cabell MF Mirra SS Beal MF Yang CC Gearing M Salvo R Mitochondrial DNA variants observed in Alzheimer disease and Parkinson disease patients Genomics 1993 17 1 171 184 10.1006/geno.1993.1299 8104867

[B44] Mancuso M Conforti FL Rocchi A Tessitore A Muglia M Tedeschi G Panza D Monsurro M Sola P Mandrioli J Could mitochondrial haplogroups play a role in sporadic amyotrophic lateral sclerosis? Neurosci Lett 2004 371 2-3 158 162 10.1016/j.neulet.2004.08.060 15519748

[B45] Czarnecka AM Krawczyk T Zdrozny M Lubinski J Arnold RS Kukwa W Scinska A Golik P Bartnik E Petros JA Mitochondrial NADH-dehydrogenase subunit 3 (ND3) polymorphism (A10398G) and sporadic breast cancer in Poland Breast Cancer Res Treat 2010 121 2 511 518 10.1007/s10549-009-0358-5 19266278

[B46] Setiawan VW Chu LH John EM Ding YC Ingles SA Bernstein L Press MF Ursin G Haiman CA Neuhausen SL Mitochondrial DNA G10398A variant is not associated with breast cancer in African-American women Cancer Genet Cytogenet 2008 181 1 16 19 10.1016/j.cancergencyto.2007.10.019 18262047 PMC3225405

[B47] Mosquera-Miguel A Alvarez-Iglesias V Carracedo A Salas A Vega A Milne R de Leon AC Benitez J Is mitochondrial DNA variation associated with sporadic breast cancer risk? Cancer Res 2008 68 2 623 625 author reply 624 10.1158/0008-5472.CAN-07-2385 18199560

[B48] Datta S Majumder M Biswas NK Sikdar N Roy B Increased risk of oral cancer in relation to common Indian mitochondrial polymorphisms and Autosomal GSTP1 locus Cancer 2007 110 9 1991 1999 10.1002/cncr.23016 17886251

[B49] Zhang JH Zhang Y Herman B Caspases, apoptosis and aging Ageing Res Rev 2003 2 4 357 366 10.1016/S1568-1637(03)00026-6 14522240

[B50] Xu L Hu Y Chen B Tang W Han X Yu H Xiao C Mitochondrial polymorphisms as risk factors for endometrial cancer in southwest China Int J Gynecol Cancer 2006 16 4 1661 1667 10.1111/j.1525-1438.2006.00641.x 16884381

[B51] Suissa S Wang Z Poole J Wittkopp S Feder J Shutt TE Wallace DC Shadel GS Mishmar D Ancient mtDNA genetic variants modulate mtDNA transcription and replication PLoS Genet 2009 5 5 e1000474 10.1371/journal.pgen.1000474 19424428 PMC2673036

[B52] Park JS Sharma LK Li H Xiang R Holstein D Wu J Lechleiter J Naylor SL Deng JJ Lu J A heteroplasmic, not homoplasmic, mitochondrial DNA mutation promotes tumorigenesis via alteration in reactive oxygen species generation and apoptosis Hum Mol Genet 2009 18 9 1578 1589 10.1093/hmg/ddp069 19208652 PMC2733816

[B53] Hertzberg M Mickleson KN Serjeantson SW Prior JF Trent RJ An Asian-specific 9-bp deletion of mitochondrial DNA is frequently found in Polynesians Am J Hum Genet 1989 44 4 504 510 2929595 PMC1715592

